# Replicative manufacturing of metal moulds for low surface roughness polymer replication

**DOI:** 10.1038/s41467-022-32767-2

**Published:** 2022-08-27

**Authors:** Sebastian Kluck, Leonhard Hambitzer, Manuel Luitz, Markus Mader, Mario Sanjaya, Andreas Balster, Marcel Milich, Christian Greiner, Frederik Kotz-Helmer, Bastian E. Rapp

**Affiliations:** 1 NeptunLab, Laboratory of Process Technology, Department of Microsystems Engineering (IMTEK) University of Freiburg, Georges-Köhler-Allee 103, Freiburg, 79110 Germany; 2 Glassomer GmbH, Georges-Köhler-Allee 103, Freiburg, 79110 Germany; 3 Gemeinnützige KIMW Forschungs-GmbH Lutherstraße 7, Lüdenscheid, Germany; 4 Institute for Applied Materials (IAM) Karlsruhe Institute of Technology (KIT) Kaiserstraße 12, Karlsruhe, Germany; 5 Freiburg Materials Research Center (FMF), Albert Ludwig University of Freiburg, Stefan-Meier-Straße 21, Freiburg, 79104 Germany; 6 Freiburg Center of Interactive Materials and Bioinspired Technologies (FIT), Albert Ludwig University of Freiburg, Georges-Köhler-Allee 105, Freiburg, 79110 Germany

**Keywords:** Design, synthesis and processing, Mechanical engineering

## Abstract

Tool based manufacturing processes like injection moulding allow fast and high-quality mass-market production, but for optical polymer components the production of the necessary tools is time-consuming and expensive. In this paper a process to fabricate metal-inserts for tool based manufacturing with smooth surfaces via a casting and replication process from fused silica templates is presented. Bronze, brass and cobalt-chromium could be successfully replicated from shaped fused silica replications achieving a surface roughnesses of R_q_ 8 nm and microstructures in the range of 5 µm. Injection moulding was successfully performed, using a commercially available injection moulding system, with thousands of replicas generated from the same tool. In addition, three-dimensional bodies in metal could be realised with 3D-Printing of fused silica casting moulds. This work thus represents an approach to high-quality moulding tools via a scalable facile and cost-effective route surpassing the currently employed cost-, labour- and equipment-intensive machining techniques.

## Abstract (web summary)

Production of tools for polymer replication in the field of optical applications is still time-consuming and cost-intensive. Here the authors develop an efficient metal casting process, and demonstrate manufacturing of structures of complex shapes with a surface roughness of few nanometres.

## Introduction

Tool based manufacturing (TBM) is the process of choice when it comes to cost-effective mass production. Even high-precision components such as cell phone camera lenses, Fresnel lenses or micro-diffusers^1,2^ with tight tolerances must be manufactured in large quantities at affordable costs. This requirement profile leaves very little choice in the manufacturing procedures and can only be realised by TBM^3,4^. Most prominently, injection moulding has emerged as the de facto gold standard for high-throughput manufacturing of complex-shaped components with a high standard of quality^5^. Among all, tools with highly polished moulding surfaces are of particular interest due to their ability to produce high-quality components of optical quality at relevant scalability and costs. However, their manufacturing is complex and expensive and remains the main bottleneck^6^. Today, moulding tools for TBM are mainly produced by subtractive machining such as drilling, turning, milling and polishing^7,8^. These procedures are time- and material-intensive and do not scale well^8,9^. To produce moulds with optical surfaces, ultra-precision machining is usually required, including diamond turning and polishing of surfaces well into the nanometre surface roughness range^7^. This limits the applicability of TBM and makes moulding tool prototyping extremely challenging. Depending on the quality, even simple moulding tools can range from thousands to tens of thousands of euros in cost^9^ with the actual manufacturing process easily spanning weeks, depending on its size, complexity and the required surface quality^8^. If micrometre or even sub-micrometre resolutions are required, electroplating is usually the method of choice. In this process, prefabricated templates shaped, e.g., via a photolithography, are copied into a hard metal substrate which can withstand the stresses of the forming process^8^, while providing surfaces of optical quality. The decisive disadvantages of electroplating are slow growth rates, 12 µm/h^10^ are not unusual for nickel coatings, and the limited freedom of design for moulding tools with significant variations in dimensions. Various attempts have been presented to enable faster and more convenient generation of moulding tools, a field commonly known as rapid tooling or direct tooling. Several techniques have been presented to structure a preform of the moulding tool via generative techniques such as, e.g., selective laser sintering (SLS)^11^ or laser beam machining (LBM)^12^. Achievable surface roughness values of these techniques are in the range of R_a_ 2–40 µm^13–15^, still requiring time-consuming and expensive post-processing. The generated preform moulding tool is then post-processed using classical machining techniques, therefore saving material and overall processing time. So far, rapid prototyping for TBM is considered viable only in selected applications and is generally not considered a scalable alternative to the classical manufacturing techniques for moulding tools.

In this work, we propose a different approach in which the moulding tool itself is generated by a moulding process, i.e., the tool is generated by metal casting from a replication template. Metal casting is a long-established technology, but it has proven difficult for high-resolution casts, as the choice of potential materials for replication template with sand casting is the most common method for the use above 1000 °C. If finer surface details are required, high temperature silicone^16^ is often the material of choice. Although structures in the micrometre range^17^ and surface roughness in the sub-micrometre range^18^ can be achieved, this process requires low-melting metals^16,19^ or special alloys^20^, as the silicone will degrade at high temperatures. The need to use low melting alloys thereby limits the mechanical stability of the moulding tool significantly. We reasoned that it should be possible to directly cast relevant tooling materials, such as cobalt-chromium, if a technology for manufacturing high-temperature resistive and high-resolution template structures is available. In this paper, such templates are made directly from fused silica glass using so-called Glassomer nanocomposites which we previously described^21^. These nanocomposites are converted into fused silica components by thermal debinding and sintering, resulting in high-temperature stable pure fused silica templates. The nanocomposites can be processed by stereolithography, 2-photon polymerisation, lithography, injection moulding or casting^21–24^. We have previously demonstrated, that a wide variety of techniques can be used to structure these nanocomposites at high resolution yielding optical surfaces via inexpensive, fast and flexible processes.

In this work we demonstrate that using these fused silica templates, metal moulds of high quality can be obtained featuring structures in the single-µm range and surface roughness values of 8 nm (R_q_) without post-treatment. The production time for a metallic mould inserts with this process requires less than 36 h allowing fast tool replacement as well as frequent design iterations (for further information see supplementary section). The fabricated moulding tools can be used in conventional high-throughput injection moulding process without limitations. As this process workflow effectively generates a moulding tool by a replication process, multiple fused silica replications can be generated from the same master structure thus rendering the common concerns in tool calculation (per-tool manufacturing cost, wear, yield-per-tool, etc.).

## Results

### Replicative metal moulding process

**Fig. 1:**
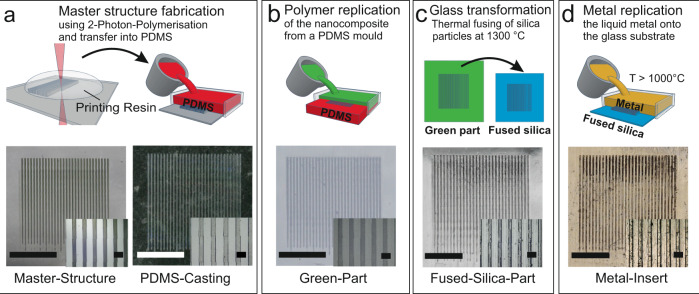
Process from the master structure to the metal replication. **a** The master (positive) structure is fabricated using 2-photon-polymerisation before being copied into polydimethylsiloxane (PDMS) via casting (negative) (scale bar: 5 mm, magnified view scale bar: 500 µm). **b** Fused silica part (positive) fabrication, by casting silica nanocomposite onto the created PDMS-Replication mould and curing it using UV-Light (scale bar: 5 mm, magnified view scale bar: 500 µm). **c** After debinding and sintering, a fully-dense and transparent fused silica replication structure is obtained (positive) (scale bar: 4 mm, magnified view scale bar: 400 µm). **d** Casting of metals against the sintered fused silica replication structure using bronze metal (negative) (scale bar: 4 mm, magnified view scale bar: 400 µm).

The production of a metal replica using our process consists of four steps: master structure fabrication, replication using the Glassomer nanocomposite, glass transformation via heat treatment of the nanocomposite and finally metal casting. Figure 1 illustrates the workflow schematically. The production of a master structure requires a free shaping method with an optical surface finish. We fabricated the master structure using 2-photon polymerisation, which is a 3D printing technology capable of printing photoresins with a resolution of down to 100 nm^25,26^ and a surface roughness in the single nanometre range^23^ (see Fig. 1a). The printed template is subsequently replicated into polydimethylsiloxane (PDMS) (see Fig. 1a). The PDMS is capable of casting features down to 500 nm^27^ and is transparent to light down to 280 nm. As illustrated in Fig. 1b, the liquid nanocomposite is poured on the PDMS-Replication mould and cured by UV light at a wavelength of 365 nm, resulting in the so-called “green part”. If necessary the green part can be further post processed using conventional subtractive polymer shaping technologies^28^. The green part is subsequently converted into transparent fully-dense fused silica glass via thermal debinding and sintering at a maximum temperature of 1300 °C as previously described^28^ (see Fig. 1c). The Glassomer L50 nanocomposite has a solid loading of 50 vol% which results in an isotropic linear shrinkage of 20.6% during the sintering process. For the metal casting, the fused silica replication is embedded in a phosphate-bonded embedding material (see Fig. 1d). Before casting, the melting chamber is flushed twice with nitrogen. The melting of the metal takes place under vacuum (10^−1 ^bar) preventing the formation of oxide layers which can lead to defects in the casted metal surface. While pouring the liquid metal, a nitrogen overpressure of 3 bar is generated in the casting chamber, which ensures conformal replication from the embedded fused silica replication.

### Replication quality and characterization

**Fig. 2:**
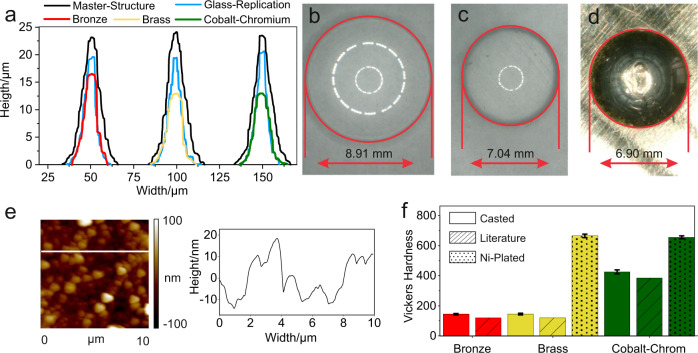
Characterization of the replicated metal moulds. **a** White-light interferometry measurement of the generated metal inserts for bronze (red), brass (yellow), and cobalt-chromium (green). **b** Optical lens master structure which was used to characterize the overall shrinkage during the process. **c** Fused silica replication of the optical lens. **d** Resulting cast bronze metal lens (negative). **e** AFM-Measurement of an unstructured casted bronze insert with a surface roughness of only R_q_ 8.0 nm. **f** Comparison of Vickers hardness values of manufactured samples of bronze (error bar standard deviation *n* = ±4 HV), brass (error bar standard deviation *n* = ±5 HV for casted and *n* = ±11 HV for nickel plated) and cobalt-chromium (error bar standard deviation *n* = ±9 HV for casted and *n* = ±13 HV for nickel plated) in pristine form and after nickel electroplating. The error bars were determined using the standard deviation of measured data, 10 measurements were carried out in each case.

We have successfully used this process for the replication of high-temperature melting metals such as bronze (1050 °C), brass (1020 °C) and cobalt-chromium (1440 °C). All of these temperatures are below the softening point of fused silica, which is 1665 °C^29^. In terms of processing properties, bronze offers very good castability at moderate melting temperatures. Furthermore, bronze is relatively corrosion-resistant and has a high thermal conductivity which makes it a material of choice for variothermal injection moulding^8,30^. Similarly, brass has good processing properties but can also be nickel-plated without pretreatment, which results in a considerable increase in hardness^31^. The cobalt-chromium alloy was chosen as a casting material because of its significantly higher hardness^32^. All three metals could be replicated from the sintered fused silica replication and demoulded to form injection-moulding compatible metal inserts. No release agent was necessary to remove the metal replications from the fused silica mould, as the metal does not bond with the fused silica components. The fused silica moulds were used only once for the metal casting, this was to ensure that a consistent quality of the metal replications could be achieved. Using high-temperature metals is of great importance for the subsequent injection moulding process since these can withstand both, the repeated temperature changes and due to their higher mechanical strength and the stresses of the moulding process. In order to determine the minimum feature resolution for each metal type, lines-and-space structures were produced and replicated using the described method (see Fig. 2). The lines are tapered, having a width between 30 µm (bottom) and 3 µm (top) and a height of 23.5 µm in the master structure. The structures were characterized in each replication step using white light interferometry (WLI). Figure 2a shows the cross-sections of the investigated master structure (black), the fused silica replication (blue) and the respective replicated metal replications (red, yellow, green). The minimum feature resolution was determined by the minimum width of the generated metal structures, measured by WLI. As shown in Fig. 2a, the minimum feature resolution is 5.2 µm for bronze, 7.5 µm for brass and 5 µm for cobalt-chromium. The difference in size between the master structure and fused silica replication is due to shrinkage during the sintering process. The measured shrinkage from the master structure to the fused silica replication, is 20.9%. This is illustrated in Fig. 2b, c, where a lens is shown as a master structure with a diameter of 8.91 mm and as a fused silica replica with a diameter of 7.04 mm. This value is in good accordance with the calculated shrinkage of 20.6% (see supplementary material). The shrinkage from the fused silica replication to the metal insert was measured to be 2.0%, 2.3%, and 1.8% for bronze, brass, and cobalt-chromium, respectively. The overall shrinkage from the master structure to the metal insert is thus 22.60%, 22.85%, and 22.45% for bronze, brass, and cobalt-chromium, respectively. It is important to note that the solidification shrinkage of metals is a complex phenomenon^33,34^ that can only be predicted to a limited extent. It is therefore necessary to assess this shrinkage experimentally. Due to the mismatch of thermal expansion coefficients of fused silica and metals, there is a risk of the fused silica being enclosed by the molten metal. As commonly employed in replication processes, demoulding chamfers can be included in the design of the master structure in order to prevent this problem. To allow for high-resolution replication of polymeric components using the metal moulds, the shrinkage during the fused silica sintering process and the metal replication process needs to be compensated in the fabrication of the master structure. Depending on the manufacturing method used to fabricate the master structure, this process related shrinkage must be taken into account as well. In order to investigate the achievable surface quality, metal inserts were prepared from an unstructured fused silica surface. Without further post-treatment, a surface roughness of 2 nm (R_q_) was measured using atomic force microscope (AFM) for the sintered fused silica components^28^. The achievable surface roughness in the casted metal inserts are measured to be only slightly higher with 8.0 nm, 9.0 nm, and 11.0 nm (R_q_) on an area of 100 µm² (see Fig. 2e, Supplementary Fig. 1a–c) for bronze, brass and cobalt-chromium, respectively. A total of nine measurements was carried out at different positions and different sized areas in order to assess the surface quality across a large lateral area. Using the WLI on a larger area (350 × 350 µm^2^), the surface roughnesses were found to be 35 nm, 28 nm and 31 nm (S_q_) for bronze, brass and cobalt-chromium, respectively. Vickers hardness was measured for all three metals to evaluate the wear resistance of the moulds during the injection moulding process^4^ (see Fig. 2f). Common, industrially employed moulds for plastic injection moulding of optical components are made from tooling steels with around 510–560 in Vickers hardness (HV)^6,8^. According to literature, values in the range of 120 HV are expected for the casted bronze^35^ and brass^36^ components. Our measurements showed a value of 151 HV for bronze and 157 HV for brass and thereby exceed the literature values slightly. For the significantly harder cobalt-chromium dental alloy, 445 HV was measured which is only slightly lower than the values expected from commercial tooling steels. As higher hardness values are desirable for injection moulding tools to extend the tool’s service life time, hardening techniques such as quenching or precipitation hardening are commonly employed which are, unfortunately, not accessible for copper-based alloys such as bronze and brass. However, an alternative is electroplating with hard nickel, a technique which achieves hardness values above 500 HV according to literature^31^. We thus coated casted brass metal moulds with a 70 µm layer of hard nickel, achieving a hardness value of 670 HV. Similar hardness values of 667 HV were achieved for nickel plated cobalt-chromium metal inserts. The Ni coating must be considered in the design, depending on the used plating technique and the layer thickness.

### Injection moulding

**Fig. 3:**
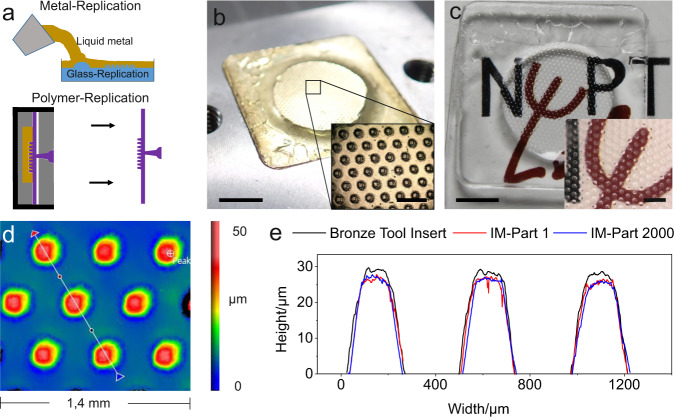
Polymer injection moulding using casted metal moulds. **a** Schematic representation of the manufacturing process of a metal insert and its use in injection moulding. **b** Close-up of the injection mould which was used as an insert (scale bar: 10 mm). The inset shows a magnification of the dot matrix structure (scale bar: 500 µm). **c** Close-up of an injection-moulded polymethylmethacrylate (PMMA) component replicated from the metal insert (scale bar: 10 mm). The inset shows a magnification of the structure (scale bar: 500 µm). **d** White-light interferometry image of the 2000^th^ PMMA component produced from the mould (IM-Part 2000) **e** Comparison of the cross-section measured using WLI of the first polymer replicated PMMA component (IM-Part 1, red) and the of the 2000^th^ component (IM-Part 2000, blue) created using the metal insert (black).

In order to assess the injection moulding compatibility of the casted metal inserts, we prepared bronze metal inserts with a dot matrix structure. The metal inserts were produced using the outlined process, followed by injection moulding in a commercial injection moulding system (Arburg Allrounder 370 S 500–100) as shown schematically in Fig. 3a. Figure 3b shows the assembled mould, used for injection moulding with polymethyl methacrylate (PMMA) (see Fig. 3c). To analyze the durability of the metal insert, more than 2000 PMMA components were produced and measured using WLI. Figure 3d shows the cross-section of the manufactured and used metal insert (black graph), the first manufactured polymer replica (red graph) and the 2000^th^ polymer replica (blue graph). The cross-section shows no notable change after 2000 replication cycles (for further information see supplementary section).

### Process variants

**Fig. 4:**
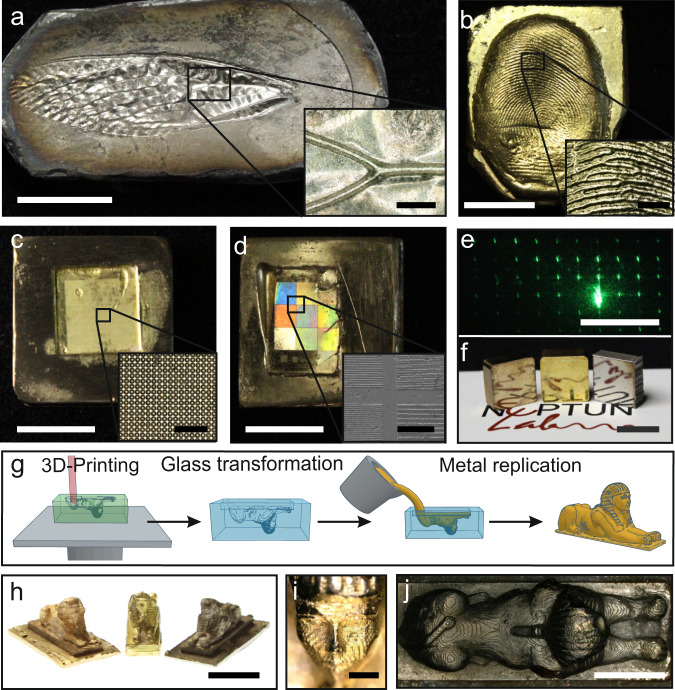
Various examples from nature and technology for the applicability of the described metal replication process. **a** Cicada wing made of a cobalt-chromium alloy (scale bar: 10 mm, magnified view scale bar: 500 µm). **b** Metal replication of a human fingerprint in brass (scale bar: 10 mm, magnified view scale bar: 500 µm). **c** Microlens array in brass with a lens diameter of 30 µm (scale bar: 10 mm, magnified view scale bar: 200 µm). **d** Bronze metal replication of different lines-and-space structures in the range of 5–25 µm in bronze showing interference effects (scale bar: 10 mm, magnified view scale bar: 100 µm). **e** Function test of a polymeric component replicated form the structure in d showing the expected diffractive far-field pattern (scale bar: 25 cm). **f** Replicated metal inserts with a mirror surface finish in bronze, brass and cobalt-chromium (scale bar: 10 mm). **g** Schematic representation of the production process of 3D-Printed Glassomer moulds for direct metal casting.  **h** Metal figurines in bronze, brass and cobalt-chromium, produced using a 3D-Printed Glassomer mould (scale bar: 10 mm). **i** Detailed view of the face of one figure, as brass metal replica (scale bar: 1000 µm). **j** Top view of the one figure, cobalt-chromium metal replica (scale bar: 5 mm). Original Sphinx design (Thing # 1404323) by Perry Engel from thingiverse.com (2016), adapted by author.

To demonstrate the applicability of the replicative metal moulding technique, various structures from nature and technology with a size from several cm to structures of a few µm were replicated (see Fig. 4 a–e). These were moulded in PDMS directly from existing objects, no master structure produced by 2-photon polymerisation was used. Bionic structures like the wing of a cicada or a human fingerprint could be directly replicated using the metal casting process (see Fig. 4a, b). Feature sizes in the range of several tens of micrometres were replicated successfully into cobalt-chromium and brass. Further we show the successful replication of refractive and diffractive microoptical elements. Figure 4c shows a microoptical lens array with lens diameters of 30 µm in brass. The sample in Fig. 4c shows a surface defect resulting from a contaminated fused silica surface. Defects of this nature can be avoided by working under clean room conditions. Figure 4d shows diffractive line-and-space structures with line widths between 5 and 25 µm in bronze. Figure 4e shows the mirror surface finish replicated from an unstructured fused silica part without post treatment after casting in bronze, brass and cobalt-chromium using the described process. A further modification of the process also allows the direct production of a 3D-Mould, from the polymer nanocomposite as schematically depicted in Fig. 4f. This allowed the direct production of moulds in the nanocomposite polymer for metal casting without the use of a master structure via 3D-Printing. After sintering, the printed mould can directly be filled with liquid metal, resulting in a metal part like shown in Fig. 4g in bronze, brass and cobalt-chromium. The lines created by the 3D-Printing process can be seen in the metal, as shown again in Fig. 4i, j.

In this paper, we demonstrated a replicative manufacturing process allowing rapid and cost-efficient production of metal inserts for polymer replication with low surface roughness, using a replication technique. We have shown that high-temperature metals like bronze, brass, and cobalt-chromium can be successfully shaped with a feature size down to 5 µm and single nanometre surface roughness. The inserts were successfully used in industrially-established polymer injection moulding instruments generating thousands of components. This process thus enables the flexible and cost-efficient production of metal inserts with low surface roughness by a replication process for tool based manufacturing like injection moulding or hot-embossing, bypassing the common problems of classical tool manufacturing such as high per-mould costs and processing times commonly associated with the high costs of classical moulding tools.

## Methods

### Materials

Glassomer L50, Glassomer SL-v2, Glassomer Developer, and Glassomer Hardener was kindly provided by Glassomer (Germany). Elastosil M4601, was purchased from Wacker (Germany). The Plaster “Pro-HT Platinum” as an embedding material, the metal alloys bronze (BR10/L) and brass (Messinggranulat Hart) were purchased from Horbach Technik (Germany). The cobalt-chromium alloy for dental purposes” Wironit extrahart” was purchased from BEGO (Germany).

### Two-photon-polymerisation

In order to produce master structures, the “NanoOne” printing system from UpNano GmbH (Austria) was used. The structures were printed on a glass substrate with the refractive index matched 2-photon resin “UpBrix”. The print was carried out using 10× magnification, a laser power of 50 mW, and a layer thickness of 5 µm.

### 3D-printing

In order to produce casting moulds directly in the polymer nanocomposite, the resin printer Prusa SL1S Speed of PRUSA (Czech Republic) was used to print. The material (Glassomer L50-SL-v2 according to the manufacturer’s specifications) for printing was kindly provided by Glassomer (Germany). The structures were directly printed onto the printing platform. The Printer was used with a wavelength of 405 nm, an exposure time of 20 s and a layer thickness of 50 µm. The printed components were developed using Glassomer developer.

### Replication of the master structures

PDMS was mixed for 1 min in a ratio of 9:1 by weight (A:B component). Entrapped air bubbles were removed using vacuum in combination with a desiccator. The master structure was fixed in a Petri dish and then moulded using PDMS in the oven at 60 °C for one hour to cure the PDMS-Replication. The cured PDMS-Replication was peeled from the master structure. Glassomer L50 was mixed with Glassomer Hardener according to the manufacturer’s specifications. Glassomer L50 was then poured onto the PDMS mould and cured by illumination at a wavelength of 320–405 nm for 2 min. After curing, the nanocomposite could be removed from the PDMS mould.

### Heat treatment and shrinkage

Thermal debinding of the cured Glassomer green parts was carried out in an ashing furnace (type AAF, Carbolite Gero, Germany) at 600 °C. The brown parts were sintered in a tube furnace (type STF16/450, Carbolite/Gero, Germany) at 1300 °C and a pressure of 5 × 10^−2^ mbar.

1 $${Y}_{s}=1-{\left(\frac{\varPhi }{{\rho }_{t}/{\rho }_{f}}\right)}^{\frac{1}{3}}$$

The theoretical shrinkage Y_s_ is calculated by Eq. (1) which depends on the solid loading Φ, the final density ρ_f_, and the theoretical density ρ_t_ of the produced part. The actual shrinkage was determined by measuring the parts in the green state, in sintered state and after metal replication using the digital microscope model VHX 6000 from Keyence (Japan).

### Fused silica embedding

In order to prepare the sintered glass components for the casting process, the components were fixed in a steel cuvette using phosphate-bonded embedding material (Pro-HT Platinum, Horbach Technik, Germany). The embedding material was mixed in a ratio of 31:100 by weight (water/powder) and poured into the prepared metal cuvette before heating at 800 °C for 2 h.

### Metal casting

For the metal casting, the prepared steel cuvette with the fused silica replication master was preheated to 200 °C to increase the form filling. The setup was then installed in the casting furnace (type M20, Indutherm, Germany). After closing the casting chamber, it was flooded with nitrogen, then a vacuum was applied and the crucible with the casting material was brought to the desired melting point (bronze 1050 °C, brass 1020 °C, Co-Cr 1450 °C). When the melting point was reached, the entire casting chamber was tilted, and the melt was allowed to flow into the steel cuvette and onto the glass body. In the tilted position, a pressure of 3 bar nitrogen was generated in the chamber. The casting furnace was left in this position until the metal body cooled.

### Characterization

The roughness was measured using an AFM of type Multimode 8 (Bruker, Germany) on an area of 10 × 10 µm as well as a WLI of type NewView 9000 (Zygo, USA) on an area of 350 × 350 µm and 860 × 860 µm (see Supplementary Fig. 1 and Table 1). All surface roughness measurements were carried out three times, at different locations. The corresponding values can be found in Supplementary Table 1. The replication limit was determined by comparing the cross-sections of a structure at different stages of the process (master, fused silica, metal) using WLI. Vickers hardness was measured using a micro Vickers hardness tester of type FALCON 608 (INNOVATEST, Netherland). The applied load was 100 mN at a loading time of 20 s.

**Table 1:** Characterization of the metal replication process

Material	Replication Limit	Roughness (R_q_ /S_q_)	Shrinkage	Hardness	Casting Temp.
Bronze	5.2 µm	8.0 ± 1 nm/35.0 ± 2 nm	22.60%	151 ± 4.5 HV	1050 °C
Brass	7.5 µm	9.0 ± 2 nm/28.0 ± 1 nm	22.85%	157 ± 5.2 HV	1020 °C
Ni-Plated Brass	—	6.0 ± 1 nm/25.0 ± 1 nm	22.85%	679 ± 11.0 HV	—
Chrom-Cobalt	5.0 µm	11 ± 1 nm/31.0 ± 3 nm	22.45%	445 ± 13.1 HV	1440 °C

Comparison of the minimum feature resolution, surface roughness, hardness, shrinkage, and casting temperature in dependence of the used metal.

## Supplementary information

Supplementary Information

## Data Availability

The authors declare that the data supporting the findings of this study are available within the paper.

## References

[1] Mayer R (2007). Precision injection molding: how to make polymer optics for high volume and high precision applications. Opt. Photonik.

[2] Sortino M, Totis G, Kuljanic E (2014). Comparison of injection molding technologies for the production of micro-optical devices. Procedia Eng..

[3] Zhang H, Zhang N, Han W, Gilchrist MD, Fang F (2021). Precision replication of microlens arrays using variotherm-assisted microinjection moulding. Precis. Eng..

[4] Fang F, Zhang N, Zhang X (2016). Precision injection molding of freeform optics. Adv. Optical Technol..

[5] Piotter V, Hanemann T, Ruprecht R, Haußelt J (1997). Injection molding and related techniques for fabrication of microstructures. Microsyst. Technol..

[6] Bäumer, S. (Hg.). *Handbook of plastic optics*. 35–62 (John Wiley & Sons, 2011).

[7] Fang FZ, Zhang XD, Weckenmann A, Zhang GX, Evans C (2013). Manufacturing and measurement of freeform optics. CIRP Ann..

[8] Menges, G., Michaeli, W. & Mohren, P. *How to Make Injection Molds*, 12–32, 85–103 (Carl Hanser Verlag GmbH Co KG, 2013).

[9] Morrow WR, Qi H, Kim I, Mazumder J, Skerlos SJ (2007). Environmental aspects of laser-based and conventional tool and die manufacturing. J. Clean. Prod..

[10] Piotter V, Holstein N, Plewa K, Ruprecht R, Hausselt J (2004). Replication of micro components by different variants of injection molding. Microsyst. Technol..

[11] Launhardt M (2016). Detecting surface roughness on SLS parts with various measuring techniques. Polym. Test..

[12] Pham DT, Dimov SS, Ji C, Petkov PV, Dobrev T (2004). Laser milling as a ‘rapid’ micromanufacturing process. Proc. Inst. Mech. Eng., Part B: J. Eng. Manuf..

[13] Gibson, I., Rosen, D. W., Stucker, B. & Khorasani, M. *Additive manufacturing technologies*. 65, 314, 458, 614 (Cham Switzerland: Springer, 2021).

[14] Kumbhar NN, Mulay AV (2018). Post processing methods used to improve surface finish of products which are manufactured by additive manufacturing technologies: a review. J. Inst. Eng. India Ser. C..

[15] Khaing MW, Fuh JYH, Lu L (2001). Direct metal laser sintering for rapid tooling: processing and characterisation of EOS parts. J. Mater. Process. Technol..

[16] Chung S, Park S, Lee I, Jeong H, Cho D (2005). Replication techniques for a metal microcomponent having real 3D shape by microcasting process. Microsyst. Technol..

[17] Schmitz GJ, Grohn M, Bührig-Polaczek A (2007). Fabrication of micropatterned surfaces by improved investment casting. Adv. Eng. Mater..

[18] Baumeister G, Ruprecht R, Hausselt J (2004). Replication of LIGA structures using microcasting. Microsyst. Technol..

[19] Cannon AH, King WP (2009). Casting metal microstructures from a flexible and reusable mold. J. Micromech. Microeng..

[20] Baumeister G, Mueller K, Ruprecht R, Hausselt J (2002). Production of metallic high aspect ratio microstructures by microcasting. Microsyst. Technol..

[21] Kotz F (2016). Liquid glass: a facile soft replication method for structuring glass. Adv. Mater..

[22] Kotz F (2017). Three-dimensional printing of transparent fused silica glass. Nature.

[23] Kotz F (2021). Two‐photon polymerization of nanocomposites for the fabrication of transparent fused silica glass microstructures. Adv. Mater..

[24] Mader M (2021). High-throughput injection molding of transparent fused silica glass. Science.

[25] Faraji Rad Z, Prewett PD, Davies GJ (2021). High-resolution two-photon polymerization: the most versatile technique for the fabrication of microneedle arrays. Microsyst. Nanoeng..

[26] Gissibl T, Thiele S, Herkommer A, Giessen H (2016). Two-photon direct laser writing of ultracompact multi-lens objectives. Nat. Photon.

[27] Williams SS (2010). High-resolution PFPE-based molding techniques for nanofabrication of high-pattern density, sub-20 nm features: a fundamental materials approach. Nano Lett..

[28] Kotz F (2018). Glassomer-processing fused silica glass like a polymer. Adv. Mater..

[29] Vass C, Smausz T, Hopp B (2004). Wet etching of fused silica: a multiplex study. J. Phys. D: Appl. Phys..

[30] Kelly AL, Mulvaney-Johnson L, Beechey R, Coates PD (2011). The effect of copper alloy mold tooling on the performance of the injection molding process. Polym. Eng. Sci..

[31] Nair S, Sellamuthu R, Saravanan R (2018). Effect of Nickel content on hardness and wear rate of surface modified cast aluminum bronze alloy. Mater. Today.: Proc..

[32] Dobbs HS, Robertson JLM (1983). Heat treatment of cast Co-Cr-Mo for orthopaedic implant use. J. Mater. Sci..

[33] Flemings MC (1974). Solidification processing. Met. Mater. Trans. B.

[34] Ravi B, Srinivasan MN (1996). Casting solidification analysis by modulus vector method. Int. J. Cast. Met. Res..

[35] Paul C, Sellamuthu R (2014). The effect of Sn content on the properties of surface refined Cu-Sn bronze alloys. Procedia Eng..

[36] Atsumi H (2010). Microstructure and mechanical properties of high strength brass alloy with some elements. MSF.

