# Supplementary material for: Replicative manufacturing of metal moulds for low surface roughness polymer replication
Source: Nat Commun. 2022 Aug 27;13:5048. doi: 10.1038/s41467-022-32767-2 (PMC9420142; doi:10.1038/s41467-022-32767-2)
Supplement: Supplementary file 1 — Supplementary Information [file 41467_2022_32767_MOESM1_ESM.pdf]

Supplementary Materials for

"Replicative Manufacturing of Metal Moulds for Low Surface Roughness Polymer Replication"

Sebastian Kluck, Leonhard Hambitzer, Manuel Luitz, Markus Mader, Mario Sanjaya, Andreas Balster, Marcel Milich, Christian Greiner, Frederik Kotz-Helmer, Bastian E. Rapp

Corresponding author

*Correspondence to: Frederik.Kotz-Helmer@neptunlab.org, ORCID: 0000-0001-8376-154X

Correspondence to: Author-9

This PDF includes:

Supplementary Figure 1 to Figure 7

Supplementary Table 1 to Table 7

Supplementary Note 1: Surface characterization with atomic force microscopy (AFM) and white light interferometry (WLI).

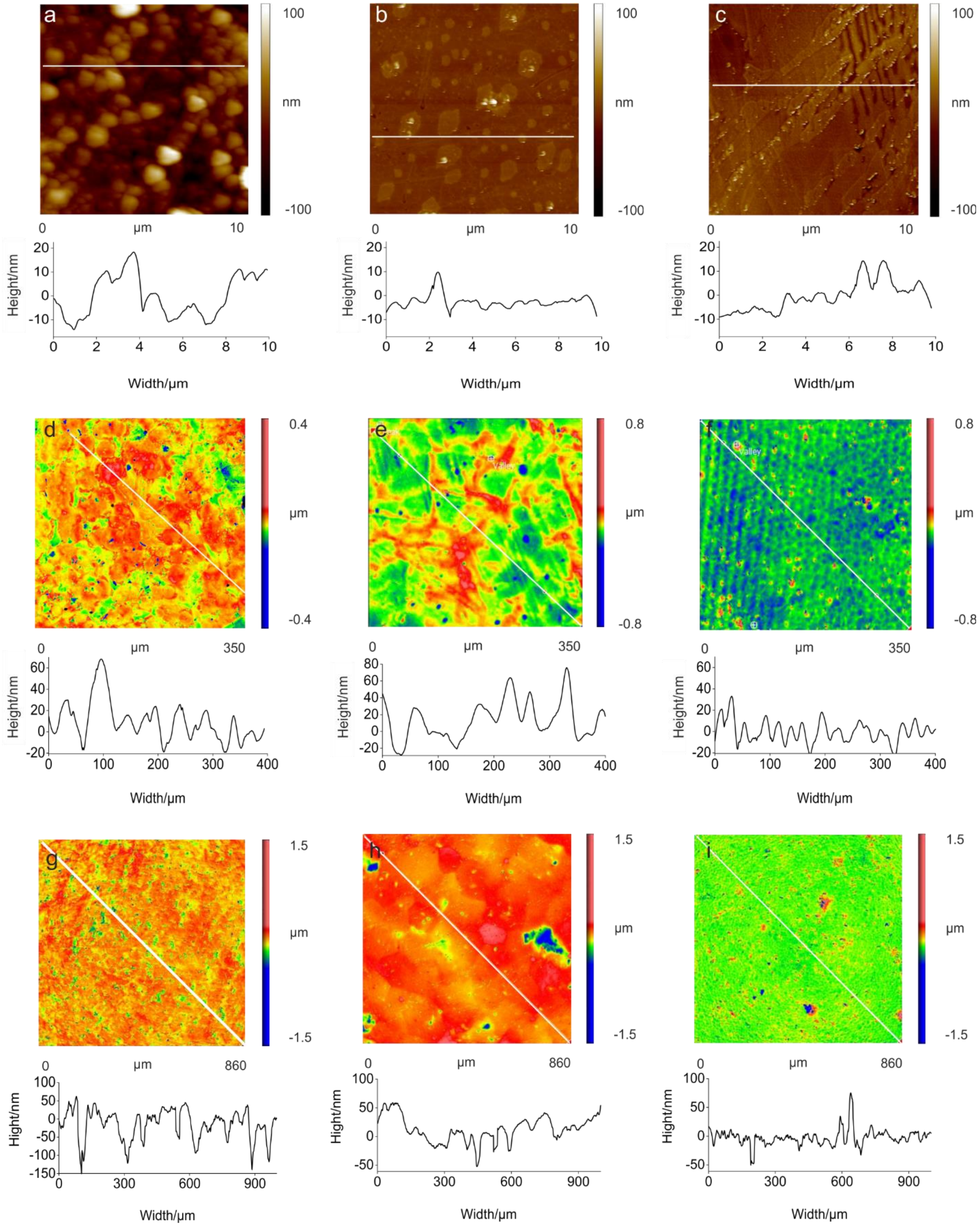

Supplementary Figure 1: **Roughness measurements of different metal casts onto flat fused silica parts using atomic force microscopy (AFM) and white light interferometry (WLI):** AFM image on an area of 10 by 10 μm^2 of bronze (a, roughness 7.5 nm), brass (b, roughness 7.2 nm) and cobalt-chromium sample (c, roughness 11.5 nm). WLI image on an area of 350 by 350 μm^2 of bronze (d, roughness 35 nm), brass (e, roughness 30 nm) and cobalt-chromium sample (f, roughness 31 nm). WLI image on an area of 860 by 860 μm^2 of bronze (g, roughness 65 nm), brass (h, roughness 53 nm) and cobalt-chromium (i, roughness 66 nm).

In order to validate the roughness measurements, the various measurements were carried out three times at different locations and different sized areas on the samples (Supplementary Table 1).

Measurement AFM 10 x 10 µm	Bronze	Brass	Cobalt-Chromium	Brass Ni-Coated
#1	8nm	7 nm	11 nm	7 nm
#2	10 nm	11nm	12 nm	6 nm
#3	7 nm	9 nm	9 nm	8 nm
Measurement WLI 350 x 350 µm				
#1	35 nm	30 nm	31 nm	26 nm
#2	34 nm	27 nm	27 nm	25 nm
#3	38 nm	29 nm	35 nm	24 nm
Measurement WLI 860 x 860 µm				
#1	65 nm	53 nm	66 nm	60 nm
#2	62 nm	63 nm	58 nm	49 nm
#3	58 nm	49 nm	58 nm	50 nm

Supplementary Table 1: **The surface roughness values measured by AFM and WLI of casted components.**

Since a replication process can (in the best case) only match the quality of the master structure, roughness measurements were made on non-structured components on the master structure, the glass replica and the final metal mold. The measurement was carried out using a white light interferometer on an area of 9 x 9 μm^2, 350 x 350 μm^2 and 860 x 860 μm^2 the results are presented in Supplementary Table 2.

	WLI 9 x 9 µm	WLI 350 x 350 µm	WLI 860 x 860 µm
Master-Structure	1 nm	6 nm	7 nm
Glass-Replication	4 nm	7 nm	12 nm
Metal-Replication	8 nm	30 nm	65 nm

Supplementary Table 2: **Comparison of the surface roughness of master-structure, the glass and metal replication.**

Supplementary Note 2: Characterization of the shrinkage during the replication

In order to be able to reproduce the shrinkage from the master structure to the finished metal component, a lens structure (see Figure 2 b-d) was produced in the metals bronze, brass and cobalt-chrome. Subsequently, the master structure, the glass replication and the metal replication were measured optically. Five measurements were made in each case. For further assessment, the profile of the lenses was measured in its entirety and the resulting lens profiles were compared. Supplementary Figure 2 shows and compares the measured lens profiles. The measured diameter of the different lens replications are shown in Supplementary Table 3.

	1	2	3	4	5	Average	Error in %
**Master in mm**	8.92	8.90	8.91	8.91	8.91	8.91	0.01
Shrinkage Master-Glass in %	21.08	20.79	21.10	21.21	20.99	21.03	0.14
**Glass in mm**	7.04	7.05	7.03	7.02	7.04	7.04	0.01
Shrinkage Glass-Bronze in %	1.99	2.13	2.28	1.85	1.70	1.99	0.20
Shrinkage Glass-Brass in %	1.99	2.13	2.56	2.28	2.56	2.30	0.23
Shrinkage Glass-Cobalt-Chromium in %	1.99	2.13	2.13	0.85	1.85	1.79	0.48
**Metal Bronze mm**	6.9	6.9	6.87	6.89	6.92	6.90	0.02
**Metal Brass mm**	6.9	6.9	6.85	6.86	6.86	6.87	0.02
**Metal Co-Cr mm**	6.9	6.9	6.88	6.96	6.91	6.91	0.03
Shrinkage Master-Bronze in %	22.65	22.47	22.90	22.67	22.33	22.60	0.19
Shrinkage Master-Brass in %	22.65	22.47	23.12	23.01	23.01	22.85	0.25
Shrinkage Master-Cobalt-Chromium in %	22.65	22.47	22.78	21.89	22.45	22.45	0.31

Supplementary Table 3: **Measured diameter of master structure, glass replication and metal replication to calculate the shrinkage during the replication process.**

In order to determine the 3-dimensional shrinkage and dimensional stability, the curvatures of the lens structures shown in Figure 2 b-d were determined and compared. We found the curvature of the lenses do remain constant overlaid only by the shrinkage-induced change in size.

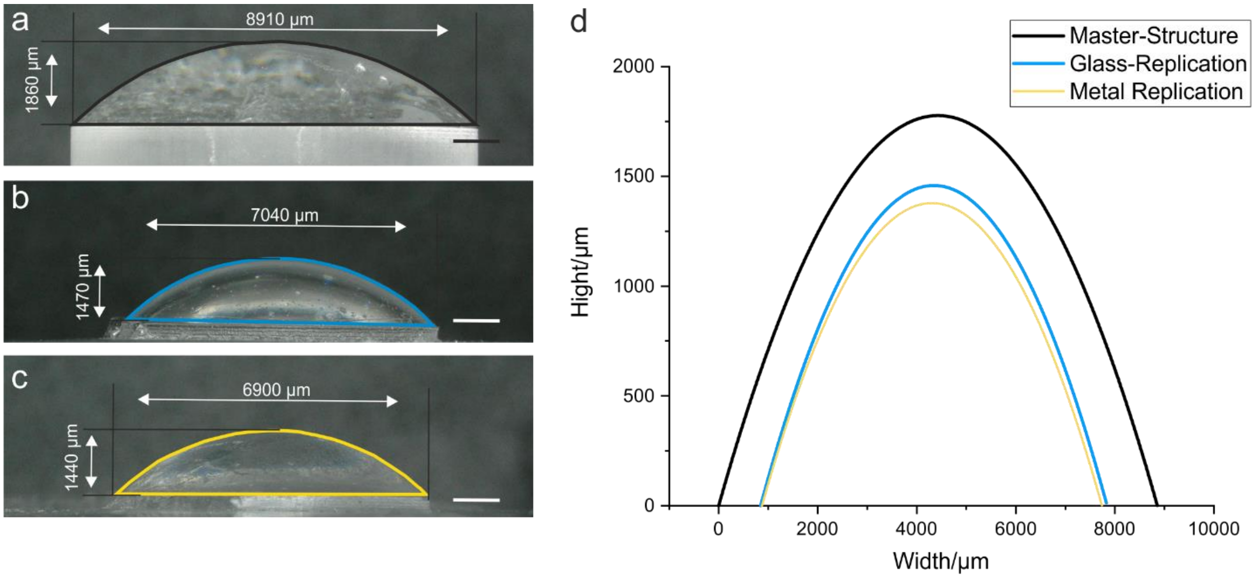

Supplementary Figure 2: **Measurement of the curvature of a macroscopic lens and its replications to determine the shrinkage**. **a** The side view of the master structure used for replication (scale bar: 1000 µm). **b** Side view of the replicated glass structure (scale bar: 1000 µm). **c** Side view of a silicone replica of the metal mould (scale bar: 1000 µm). **d** White light interferometry measurements of the curvature of the master structure, the glass replica and the silicone replica.

To determine that the shrinkage is isotropic as expected, the curvature of a lens structure and its replications were measured. 2 a-c shows the side views of these structures. In order to characterize the metal mould (negative) and compare it to the used templates a silicone replication of the metal mould was used. The measurements of the curvature are presented in Supplementary Figure 2 d, which shows that the curvature of the lens does not change due to shrinkage.

Supplementary Note 3: Characterization of polymer parts produced with injection-moulding

In order to investigate the wear and tear of the injection mould inserts, the height of the manufactured parts were measured at the beginning and at the end of the test series. Figures S3 and S4 show the measurement using WLI. Supplementary Table 4 shows the measured values of the height of the structures as well as the standard deviation and the relative standard deviation.

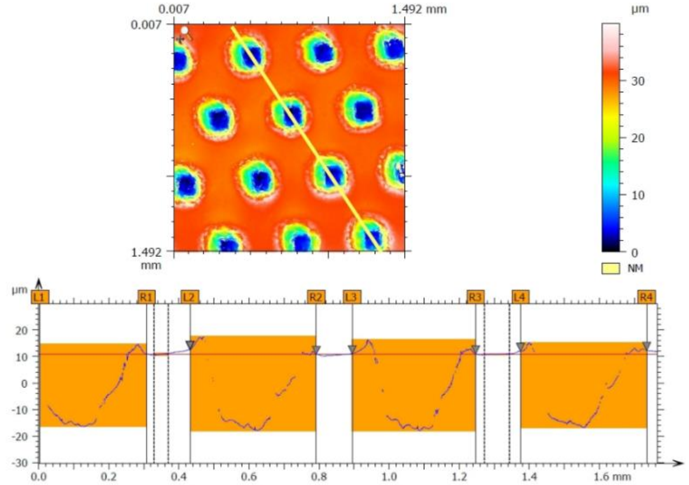

Supplementary Figure 3: **Measurement of pits on a bronze metal mould structure after one injection-moulding shot using PMMA.**

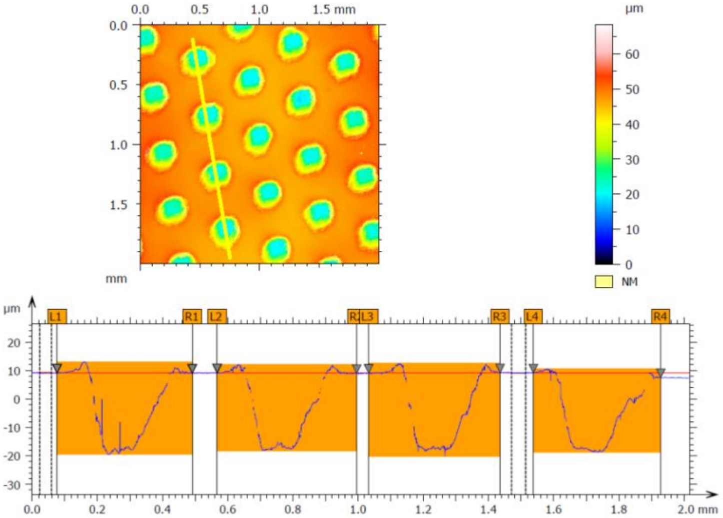

Supplementary Figure 4: **Measurement of pits on a bronze metal mould structure after 2000 injection-moulding shots using PMMA.**

Height (µm)	IM-Part 1	IM-Part 2000
Measurement #1	27.31	28.69
Measurement #2	28.79	27.28
Measurement #3	28.83	28.44
Measurement #4	27.70	27.93
Average	28.29	28.10
Standard deviation	0.77	0.62
Rel. Standard deviation	2.7%	2.2%

Supplementary Table 4: **Measurement data from the measurement of the structures IM-Part 1 and IM-Part 2000 as well as standard deviation and relative standard deviation.**

In order to demonstrate that the polymer replications in injection moulding do not have a negative effect on the mould, further samples were measured and compared using white light, the corresponding graph can be found in Supplementary Figure 5. Here it can be observed that the replications are all identical and therefore no negative influence on the injection mould is found after 2000 replications.

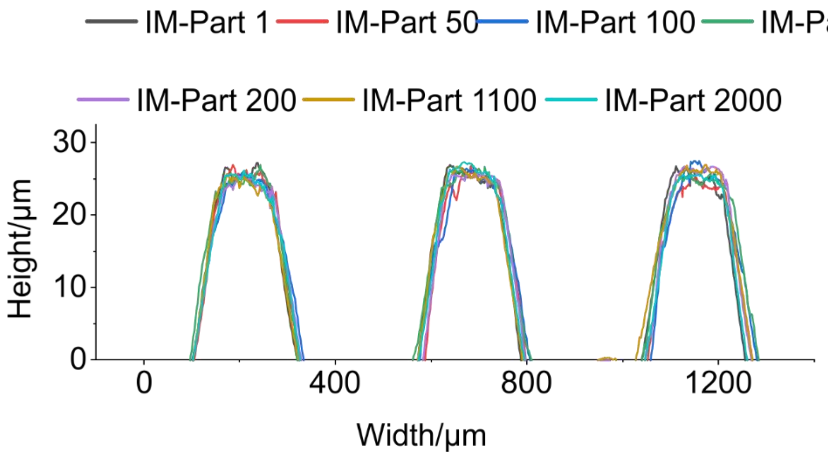

Supplementary Figure 5: **WLI measurements of different components replicated using injection moulding (IM-Part 200 = #200 of all components produced) with a tool made of bronze using the RMM-Process.**

Supplementary Note 4: Comparison of methods and process performance

A table of comparison of the technology developed within this paper to the current state of the art for the fabrication of metal tools is shown in Supplementary Table 5, including time and cost to produce a metal tool using the different processes as well as the achievable surface roughness and minimum feature size. In addition, criteria were chosen that either have an influence on the production time and cost or show a special feature of the developed process. The costs and time comparisons listed in Supplementary Table 7 are related to the quality features given in Supplementary Table 5 (Surface finish, Realistic minimum feature size).

Literature			[1–7]	[8–11]	[12–16]	[17–20]
Factor	RMM	RMM + Galvanic	CNC	Laser cutting	SLS	Galvanoforming
Scalability	++	+	+	+	+	+
Cost	++	++	--	+	+	--
Production time first mould	< 36 h	~ 36 h	~ 55 h	++	++	~ 250 h
Production time subsequent mould	< 1 h	~ 1 h	~ 55 h	++	++	~ 250 h
Post Processing	++	++	+	--	--	+
Material selection	+	+	+	++	++	0
Durability	+	++	++	+	+	++
Flexibility	++	++	-	+	++	+
Replaceability	++	++	-	-	-	-
Price is structure /quality dependent	No	No	Yes	Yes	Yes	No
Can circada wings be made?	++	++	--	-	-	+
3D capability	++	++	-	-	++	++
Surface finish	~ 8 nm (Rq)	< 8 nm (Rq)	1 nm - 1 µm (Ra)	0.5 - 4 µm (Ra)	0.2 - 60 µm (Ra)	< 10 - 100 nm (Ra)
Realistic minimum feature size	< 8 µm	< 8 µm	5 - 30 µm	3 - 40 µm	10 - 100 µm	< 1 µm
++ excellent + good 0 neutral - poor -- very poor						

Supplementary Table 5: **Comparison of relevant methods for manufacturing of injection mould tooling.**

We have calculated the manufacturing cost and time for an exemplary microlens array as shown in Figure 4 c in the main part of the manuscript and compared it to the state of the art processes shown in Supplementary Figure 3. As can be seen only RMM, CNC and galvanoforming are capable of manufacturing these microstructures. Estimated on market research costs for CNC manufacturing and galvanoforming these molds are in the range > 10k€ and 5-10k€, respectively. The manufacturing time for CNC machining and galvanoforming of these molds are 55 h[7,21,22] and 250 h[17], respectively. All subsequent molds require the same time effort and costs as the first mold.

The manufacturing time for the first tool insert using the process described in this paper is listed in Supplementary Table 6 sorted by the necessary work steps. As can be seen the overall fabrication time for the first metal mold is roughly 36 h. Since only step 7 and 8 have to be repeated for all subsequent moulds the time is tremendously reduced to only 1 h.

Step	Operation	Time
1	Master Structure production	~ 1 h
2	PDMS Replication: Replication of the master structure in PDMS	1 h
3	Polymer Replication: Replication with nano comp. from PDMS	0.25 h
4	Thermal Treatment: Thermal debinding and sintering	24 h
5	Embedding: Embedding the glass part into plaster for casting	0.5 h
6	Firing of the investment material (plaster)	8 h
7	Casting: Casting of the metal onto the embedded glass part	0.25 h
8	Post-Treatment (Milling): Milling the casted metal parts to the desired shape and diameter	0.5 h
	Total working time excluding the thermal processes and master template production	2.50 h
	Total time for subsequent molds	1 h
	Total time for preparation of mould insert using RMM	34.50 h
	Total time for preparation of a mould insert including printing of the master structure	35.50 h

Supplementary Table 6: **Display of the time required for the individual production steps around RMM processes.**

An overview of the costs for the production of a tool insert with the RMM process is shown in Supplementary Table 7. As can be seen the costs for the first metal mold can be roughly estimated to be

151.68 €. Since only the metal replication process needs to be repeated for each subsequent mold the costs for each subsequent mold is reduced to 71.20 €.

Manufacturing parameters					
Material	Brass				
Form /Structure	Lens-Array				
Weight	~ 50 g				
Surface	Low surface roughness				
Dimension	25 x 25 mm				
Material cost	Price per kg / piece	Used units	Factor		Total cost
Casting material	22.5 €	0.05 kg	1		1.13 €
Embed material	5.7 €	0.1 kg	1		0.57 €
Glass replication	1000 €	0.01 kg	1		10 €
Material overheads					
PDMS	62.6 €	0.1 kg	0.1		0.63 €
Cuvettes	12 €	1	0.05		0.6 €
Production costs	Cost / Time	Time used	Factor		Total cost
Working time	20 €/h	2.50 h	1		50 €
Production overheads	Cost per unit	Used units	Factor		Total cost
Energy in kWh	0.25 €	25 kWh	1		6.25 €
Sinter oven	25000 €	1	0.001		25 €
Debinding oven	4500 €	1	0.002		9 €
Casting oven	13500 €	1	0.001		13.5 €
Cost of services					Total cost
Post-processing					35 €
Sum					151.68 €

Supplementary Table 7: **Production costs of a component using the RMM process.**

In order to demonstrate the flexibility as well as the technical scalability of the process used, Supplementary Figure 6 shows the manufactured components when used in the injection moulding process. Supplementary Figure 6 a illustrates the assembly of the manufactured mould inserts in a designated holder. Supplementary Figure 6 b shows the corresponding steel holder and four mould inserts in the unmounted state. The molud inserts are fixed in the holder from behind and from the side with screws so that they can be moved during the injection moulding process. The mould surface of the mould inserts is covered with a film to protect them from damage during assembly. Supplementary Figure 6 c shows the assembled mould insert in the steel holder. Supplementary Figure 6 d shows the base mould used in which the holder with mould insert can be clamped. Supplementary Figure 6 e shows the base mould used together with the holder and mould insert mounted in the injection-moulding machine from Arburg. Supplementary Figure 6 f shows the machine manufacturer and the type designation of the injection moulding machine used for plastic replication. Produced parts using the casted mould insert are shown in Supplementary Figure 6 g,h,i. For the injection-moulding process PMMA was used as clear and as black colored material.

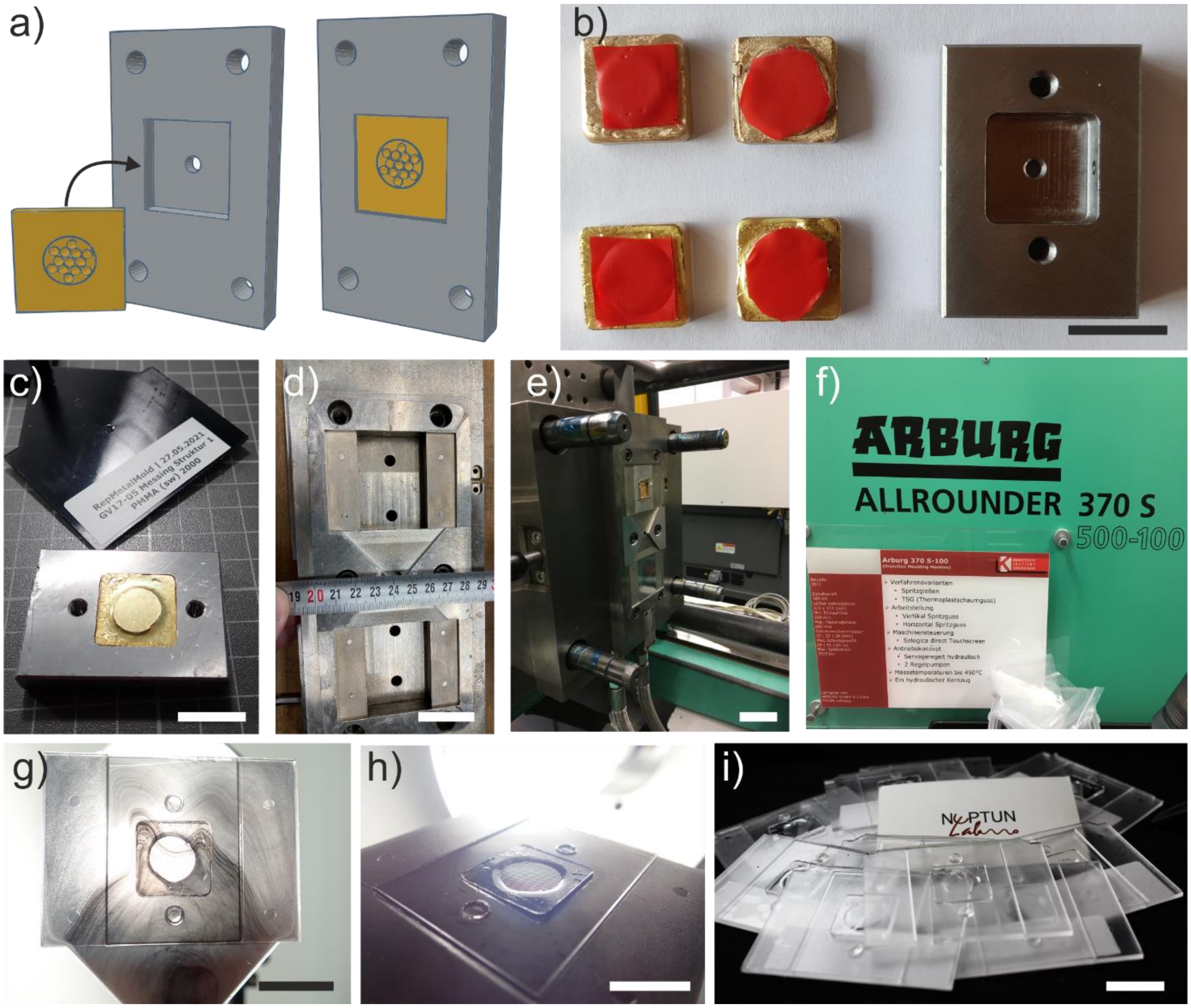

Supplementary Figure 6: **Installation and function of the manufactured mould inserts.** a) Schematic representation of an insert and its holder. b) Manufactured inserts in bronze and brass together with the steel holder, (scale bar: 20 mm). c) Brass insert, mounted in the holder, (scale bar: 20 mm). d) Base mould, in which the holder is mounted together with the insert, (scale bar: 40 mm). e) Base mould with holder and an insert mounted in the Arburg Allrounder 370 S, (scale bar: 80 mm). f) Lettering on the Arburg Allrounder 370 S injection-moulding machine. g) Manufactured injection moulded part during the transition from clear PMMA to black PMMA, (scale bar: 20 mm). h) Injection moulded replica of the insert used in black PMMA, (scale bar: 20 mm). i) Manufactured injection moulded parts made of clear PMMA, (scale bar: 20 mm).

Supplementary Note 5: Structure replication and thermal processing

In order to be able to produce smooth metal samples and to determine the minimum achievable surface roughness, unstructured fused silica components were produced. The process to fabricate the unstructured fused silica glass components is shown in Supplementary Figure 7. The nanocomposite was cast between two glass plates in a cavity, cured, demoulded and converted to fused silica glass using debinding and sintering.

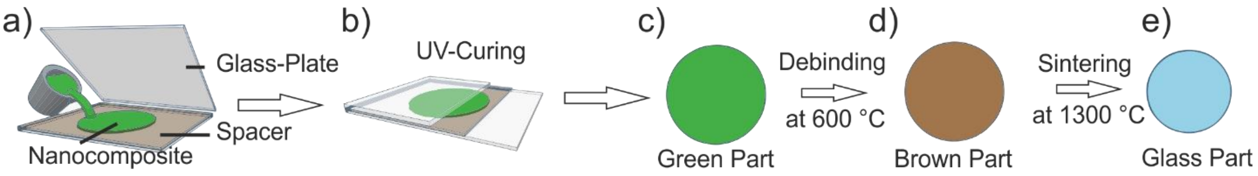

Supplementary Figure 7: **Preparation of smooth fused silica samples**. a) Schematic representation of the experimental setup for the production of smooth unstructured fused silica components. b) Curing of the nanocomposite between two glass plates. c) The resulting smooth green part. d) Debinding resulting in the so called brown part. e) Sintering resulting in a transparent fused silica glass.

Supplementary References

1. Menges, G., Michaeli, W. & Mohren, P. *How to Make Injection Molds,* 12-103 (Carl Hanser Verlag GmbH Co KG, 2013).
2. Yuan, J., Lyu, B., Hang, W. & Deng, Q. Review on the progress of ultra-precision machining technologies. *Front. Mech. Eng.* **12**, 158–180 (2017).
3. Ribeiro, J. E., César, M. B. & Lopes, H. Optimization of machining parameters to improve the surface quality. *Procedia Structural Integrity* **5**, 355–362 (2017).
4. Yan, Y., Sun, T., Liang, Y. & Dong, S. Investigation on AFM-based micro/nano-CNC machining system. *International Journal of Machine Tools and Manufacture* **47**, 1651–1659 (2007).
5. Weule, H., Hüntrup, V. & Tritschler, H. Micro-Cutting of Steel to Meet New Requirements in Miniaturization. *CIRP Annals* **50**, 61–64 (2001).
6. Egashira, K., Hosono, S., Takemoto, S. & Masao, Y. Fabrication and cutting performance of cemented tungsten carbide micro-cutting tools. *Precision Engineering* **35**, 547–553 (2011).
7. Gläbe, R. & Riemer, O. Diamond machining of micro-optical components and structures. in (eds. Thienpont, H., Van Daele, P., Mohr, J. & Zappe, H.) 771602 (2010). doi:10.1117/12.859075.
8. Ahmed, N. *et al.* Machinability of titanium alloy through laser machining: material removal and surface roughness analysis. *Int J Adv Manuf Technol* **105**, 3303–3323 (2019).
9. Teixidor, D., Ferrer, I., Ciurana, J. & Özel, T. Optimization of process parameters for pulsed laser milling of micro-channels on AISI H13 tool steel. *Robotics and Computer-Integrated Manufacturing* **29**, 209–218 (2013).
10. Pham, D. T., Dimov, S. S., Ji, C., Petkov, P. V. & Dobrev, T. Laser milling as a ‘rapid’ micromanufacturing process. *Proceedings of the Institution of Mechanical Engineers, Part B: Journal of Engineering Manufacture* **218**, 1–7 (2004).

11. *LIA handbook of laser materials processing*. (LIA, 2001).

12. Launhardt, M. *et al.* Detecting surface roughness on SLS parts with various measuring techniques. *Polymer Testing* **53**, 217–226 (2016).

13. Mendible, G. A., Rulander, J. A. & Johnston, S. P. Comparative study of rapid and conventional tooling for plastics injection molding. *RPJ* **23**, 344–352 (2017).

14. Exner, H., Regenfuss, P., Hartwig, L., Kloetzer, S. & Ebert, R. Selective laser micro sintering with a novel process. in (eds. Miyamoto, I., Ostendorf, A., Sugioka, K. & Helvajian, H.) 145 (2003). doi:10.1117/12.540730.

15. Vyatskikh, A. *et al.* Additive manufacturing of 3D nano-architected metals. *Nat Commun* **9**, 593 (2018).

16. Masuzawa, T. State of the Art of Micromachining. *CIRP Annals* **49**, 473–488 (2000).

17. Piotter, V., Holstein, N., Plewa, K., Ruprecht, R. & Hausselt, J. Replication of micro components by different variants of injection molding. *Microsystem Technologies* **10**, 547–551 (2004).

18. Lantada, A. D. *et al.* Toward mass production of microtextured microdevices: linking rapid prototyping with microinjection molding. *Int J Adv Manuf Technol* **76**, 1011–1020 (2015).

19. Shiu, P. P., Knopf, G. K., Ostojic, M. & Nikumb, S. Rapid fabrication of tooling for microfluidic devices via laser micromachining and hot embossing. *J. Micromech. Microeng.* **18**, 025012 (2008).

20. Murthy, S., Pranov, H., Pedersen, H. C. & Taboryski, R. Replication of nanopits and nanopillars by roll-to-roll extrusion coating using a structured cooling roll. *Journal of Vacuum Science & Technology B, Nanotechnology and Microelectronics: Materials, Processing, Measurement, and Phenomena* **34**, 06KM02 (2016).

21. *Handbook of plastic optics*. (Wiley-VCH, 2010).

22. Davis, G. E., Roblee, J. W. & Hedges, A. R. Comparison of freeform manufacturing techniques in the production of monolithic lens arrays. in (eds. Burge, J. H., Fähnle, O. W. & Williamson, R.) 742605 (2009). doi:10.1117/12.824451.
